# Peer-supported Open Dialogue: a qualitative study of peer practitioners’ experiences and non-peer practitioners’ perspectives on peer involvement

**DOI:** 10.1192/bjo.2025.10833

**Published:** 2025-09-19

**Authors:** Eleftherios Anestis, Tim Weaver, Jerry Tew, Sarah Carr, Corrine Hendy, Claire Melia, Katherine Clarke, Stephen Pilling

**Affiliations:** Department of Mental Health and Social Work, Faculty of Health, Social Care & Education, Middlesex University London, UK; Mental Health and Social Work School of Social Policy, University of Birmingham, UK; Department of HSPR, Institute of Psychiatry, Psychology and Neuroscience, King’s College London, UK; Nottinghamshire Healthcare Foundation Trust, Nottingham, UK; Department of Clinical Educational and Health Psychology, Division of Psychology and Language Sciences, University College London, UK

**Keywords:** Depressive disorders, randomised controlled trial, psychosocial interventions, community mental health teams

## Abstract

**Background:**

Peer-supported Open Dialogue (POD) integrates peer practitioners within mental health teams, fostering a collaborative, person-centred and social network approach to care. Although peer practitioners are increasingly involved in Open Dialogue, the role of peer practitioners within such teams remains underexplored.

**Aims:**

This study aimed to explore (a) the experiences of peer practitioners working within Open Dialogue teams in the Open Dialogue: Development and Evaluation of a Social Intervention for Severe Mental Illness trial, and (b) the perspectives of non-peer Open Dialogue practitioners regarding peer involvement. Our further objectives were to understand the nature, degree and perceived impact of peer practitioner involvement in Open Dialogue.

**Method:**

A qualitative study was conducted using semi-structured interviews and joint interviews with peer practitioners (*n* = 9). Additionally, excerpts from 11 interviews and 4 focus groups (*n* = 18), in which non-peer practitioners discussed peer practitioners’ contributions in Open Dialogue, were analysed. Thematic analysis was employed to identify key themes.

**Results:**

Three themes were developed. The first focuses on the perceived influence of peer practitioners on Open Dialogue network meetings; the second explores the opportunities and challenges of working as a peer practitioner in Open Dialogue, while the third details the perceived impact of peer practitioners on team and organisational culture.

**Conclusions:**

Open Dialogue’s emphasis on a flattened hierarchy facilitates the integration of peer practitioners, enabling them to contribute meaningfully to network meetings and team culture. Despite the overall positive experiences, peers still faced common challenges faced by those in other services, such as low pay and occasional instances of a compromised, flattened hierarchy.

Open Dialogue is an approach to mental healthcare, originating in Finland, that proposes the reorganisation of services and delivery of care based on seven principles: immediate help, social network perspective, flexibility and mobility, responsibility, psychological continuity, tolerance of uncertainty and dialogism.^1,2^ The approach views psychiatric symptoms as meaningful and adaptive responses to traumatic events, as opposed to the brain- and symptom-centric approach of the biomedical model.^3^ Care in Open Dialogue is delivered through ‘network meetings’, usually involving members of the social network of the ‘person with lived experience’^a^ (PWLE) (family and/or informal support), and two Open Dialogue practitioners who ideally remain consistent throughout care. Network meetings are characterised by a flattened hierarchy wherein all contribute equally to a dialogue that aims to mobilise the network’s resources to understand and manage a mental health crisis and achieve and maintain recovery.^3^ Unlike conventional models of care, where roles and responsibilities between care providers and recipients are prescribed and the distribution of power is unequal, flattened hierarchy in Open Dialogue encourages practitioners to adopt a ‘not knowing’ approach.^3^ It calls for practitioners to shift from ‘doing to’ to ‘being with’ networks, placing more emphasis on empathy and empowering networks to take a more active role in their care.^4^

In the context of an internationally acknowledged imperative for transforming mental health services towards person-centred, recovery- and network-oriented care,^5^ Open Dialogue services are increasingly being implemented worldwide.^6^ Given the need to operate Open Dialogue within healthcare systems widely divergent from Open Dialogue’s origins in rural Scandinavia, implementation has been characterised by adaptation and the addition of other care elements. One example is the addition of peer practitioners’^7^ involvement.^b^

## Peer support in mental health services

In mental healthcare, peer workers draw from their lived experience of mental health difficulties to provide emotional and/or practical support to PWLE.^10^ Although there are several models of peer support, most share similar values such as an emphasis on building equal power relationships based on shared experiences, the reciprocity of helping and learning and using experiential rather than taught knowledge.^11^ A recent systematic umbrella review^12^ on the effectiveness and implementation of peer support in mental health services suggested that, despite mixed meta-analytic results, peer support may improve recovery, self-efficacy and stigma-related outcomes, as well as reduce the risk of hospitalisation in adults with severe mental illness. The authors argue that differences in the implementation of peer support may explain the mixed evidence regarding its effectiveness, with adequate training and supervision, a recovery-focused and supportive workplace culture and strong leadership being associated with more positive outcomes. For those providing peer support, the benefits include improved confidence and self-esteem, social contacts, overall wellness and recovery and professional development.^12^ Conversely, negative experiences related to poor pay and emotional stress generated by the work itself,^13–16^ and negotiating the dual identity of a person with lived experience to support others while being a staff member adherent to service protocols.^16,17^

## Peer support in Open Dialogue

The emphasis on developing recovery-oriented mental healthcare services has led to a growth in peer support services worldwide. Like peers in more conventional models of healthcare, those in Open Dialogue can have various roles, from being formally trained in the approach and facilitating network meetings to offering support outside of network meetings.^7^ In the UK, peer practitioners trained in Open Dialogue have been formally integrated within therapeutic teams. This has been termed Peer-Supported Open Dialogue (POD), highlighting the perceived critical role of peer practitioners in service delivery.^18^ Underpinning POD is the bringing together of practice principles derived from Intentional Peer Support^19^ with those of Open Dialogue.^20^

The clinical and cost-effectiveness of POD is being evaluated in a large, multi-site, randomised controlled clinical trial called Open Dialogue: Development and Evaluation of a Social Intervention for Severe Mental Illness (ODDESSI).^21^ Although previous evaluations of Open Dialogue have shown promising outcomes, unlike the ODDESSI trial, these have not employed robust experimental methods.^22^ Moreover, despite the increased involvement of peers in Open Dialogue services, there is limited published evidence about their experiences and the nature of their work.^7^

It is important to acknowledge that, by virtue of the common training received by all Open Dialogue practitioners in the ODDESSI trial, peer practitioners and those from other backgrounds such as social work, medicine, nursing or care coordination may be considered to have had equivalent competency in the delivery of Open Dialogue. Indeed, the intention behind the design of the Open Dialogue intervention delivered in the trial was that all practitioners work within a flattened hierarchy that diminishes traditional professional identities. However, while peer practitioners drew on their lived experience (and were explicitly encouraged to by practising self-disclosure), others with disciplinary training underwent a ‘transformational’ experience when undergoing Open Dialogue training in which some established clinical practices had to be ‘un-learnt’.^23^

The aims of this study were to explore (a) the experiences of Open Dialogue-trained peer practitioners within the ODDESSI trial and (b) the perspectives of non-peer Open Dialogue practitioners about working with peers. Our further objectives were to understand the nature, degree and perceived impact of peer practitioner involvement in Open Dialogue.

## Method

### Study design

We conducted a qualitative process evaluation that aimed to contextualise and interpret the ODDESSI trial outcomes by exploring the experiences of the PWLE and the practitioners who participated in the trial. For this paper, we analysed data from semi-structured interviews and focus groups with peer practitioners and other Open Dialogue practitioners in non-peer roles within the multidisciplinary teams (MDT) – for convenience, referred to henceforth as ‘non-peer practitioners’). Topic guides were developed following a review of the literature on Open Dialogue, and included questions relevant to the process evaluation of the trial (supplementary material available at https://doi.org/10.1192/bjo.2025.10833).

Ethics statement: the authors assert that all procedures contributing to this work comply with the ethical standards of the relevant national and institutional committees on human experimentation, and with the Helsinki Declaration of 1975 as revised in 2013. All procedures involving human subjects/patients were approved by the Health Research Authority and the Health and Care Research Wales committee (project ID: 259468).

### Participants and recruitment

Staff who had completed Open Dialogue training and worked as Open Dialogue practitioners in one of the six National Health Service sites taking part in the ODDESSI trial were eligible for inclusion in the study. Potential participants were approached via email, sent an information sheet and asked to give informed consent to an interview. Verbal consent was witnessed and formally recorded.

During the data collection period (mainly 2020 to 2021), 11 peer practitioners worked across the 5 study centres. We conducted six individual interviews and two joint interviews; one of those in a joint interview also took part in an individual interview. Hence, the sample included 9 of the 11 peer practitioners. Sampling from a population of 42 Open Dialogue practitioners employed during the study period, 30 interviews and focus groups was conducted with non-peer practitioners as part of the process evaluation. This analysis draws upon the sections of 11 interview and 4 focus group transcripts (*n* = 18, three participants took part in both an individual interview and a focus group), where peer practitioners’ contribution in Open Dialogue was discussed. Focus groups comprised between three and five participants, but our sample for this study includes only those participants who discussed their perceptions and experiences of working with peer practitioners during these focus groups.

### Data analysis

Interviews and focus groups were audio recorded and transcribed verbatim. Thematic analysis, as described by Braun and Clarke.^24,25^ was used to analyse the data. Data familiarisation was reached after reading each transcript and interview excerpts once, before proceeding to analysing the data. Data were then assigned codes by E.A. following an inductive approach, with the coding framework refined through ongoing discussions between E.A. and T.W. Codes were grouped to form potential themes, with the final themes developed through discussion within the research team. Because of the limited pool of Open Dialogue practitioners involved in the trial, demographic information is not presented to maintain the anonymity of participants.

## Results

Three themes were developed. The first focuses on the perceived influence of peer practitioners on Open Dialogue network meetings. The second explores the opportunities and challenges of working as a peer practitioner in Open Dialogue, while the third details the perceived impact of peer practitioners on team and organisational culture.

### Theme 1: peer practitioners’ influence on network meetings

#### Self-disclosure

During the interviews, peer practitioners were asked to reflect on their experience with dialogic practice, and non-peer practitioners were asked to share their perspectives on what peers can bring to Open Dialogue. One of the issues most discussed was the use of self-disclosure in network meetings. Although Open Dialogue encourages self-disclosure for all MDT members, peer practitioners believed that their experiences were qualitatively different from those occasionally shared by their colleagues. Having been given a mental illness diagnosis, received mental health services and achieved and maintained recovery, peers felt that sharing their experiences with networks was particularly useful. Self-disclosure was not seen as a straightforward narrative act, but rather as a process that required reflection and empathy. Peer practitioners perceived self-disclosure as a ’balancing act’ (Peer Practitioner 1, PP1), requiring careful consideration of when and how much to disclose, being mindful of the need to ensure that the information would be relevant and helpful while maintaining focus on the person at the centre of concern:

‘I think you’ve got to think about why you’re sharing, how much you should share. I think at first I was almost just like splurting it out, but I think if it’s helpful to the network then I think it’s worth sharing, but sometimes, I think you have to pause and reflect a little before you share. … I don’t want to make it about me, because it’s kind of this is your network meeting. I think that’s the tightrope with Open Dialogue.’ (PP2)

Although concerns about the risk of self-disclosure shifting the focus of network meetings onto peers were occasionally reported, both peer and other participants felt that peers’ self-disclosure during network meetings could be impactful in various ways. By sharing their personal experiences, peers could help bring equality to the meetings, a sense of normalisation and validation. Clients witnessing peers’ self-disclosure could realise that ‘they are not alone’, relate with peers and start developing a relationship based on mutual understanding. Moreover, via self-disclosing, peers could bring what one peer practitioner (PP3) called the ‘missing part’ in mental healthcare: the ‘embodied experience’. Peers described how they could discuss with networks how certain medications made them feel, what helped them cope with a crisis or how they felt when they experienced certain symptoms. Their perspectives could ‘open up’ (PP4) the conversation and invite other members of the network to share their own perspectives. Especially when self-disclosure focused on recovery, participants believed that their stories could offer networks hope, reassurance and confidence that recovery is achievable:

‘I guess it’s to offer hope and reassurance that they can recover in some way, whatever that recovery may be. Obviously using my own lived experience of help, dealing with distress and discomfort and how I’ve achieved that and how I recovered, sharing that and then kind of being a support for them, a pair of ears, try and give them confidence.’ (PP2)

#### Sensitivity, understanding and relationship building

Having lived through similar mental health difficulties, peers seemed to have an enhanced ability in regard to understanding clients’ emotional states and narratives. One peer discussed how they sometimes helped networks ‘put things into words’ (PP6) and develop a conversation, especially when the person at the centre of concern was experiencing a crisis and could not verbalise their thoughts and feelings. Similarly, thanks to their own personal experiences, another peer felt that they were able to communicate better with people who were going through a manic episode. Via an enhanced understanding of, and sensitivity to, how PWLE might feel and think when they receive support, he was able to tailor his approach to responding to clients’ needs:

‘I think I can communicate better with people with mania, better than most people can. They just have an issue talking and I don’t seem to annoy people as much as other people do when talking to people with mania. Maybe knowing when to give space and when to be firm. But there is one specific example with someone and the way he was using language was quite similar to the way I did in a manic state. I think he was getting frustrated about people not just giving yes and no answers and being very indefinite with their language.’ (PP4)

Peers also believed that, thanks to their lived experience, they often felt more comfortable in discussing sensitive topics with networks. These could include drug problems, hearing voices or the side-effects of medication that can impact one’s sexual life or body image. By validating clients’ experiences and concerns via sharing their personal stories, peers felt they could assist in reducing the stigma surrounding these topics and help clients feel listened to and understood. Having been ‘on the wrong side of it’ (PP2), participants were sensitive about not devaluing clients’ experiences but allowing for their voice to be genuinely heard so that a trusting therapeutic relationship could be developed:

‘You know when people are in immediate crisis, and being there myself, it’s very hard to trust, it’s very hard to understand true from false. It’s very hard to kind of build relationships with people, because actually sometimes when you are translating some of the things that are happening to you, you are then being told that these things are not happening to you. So now I try to just sit there with a person, allowing them that safe space to share and truly listen to them. This helps people feel that they can trust us and that we can all work together to manage these issues.’ (PP3)

Similarly, non-peer practitioners often expressed the belief that peers had a unique ability in establishing a connection with PWLE, thanks to their common experiences. One mentioned that building rapport can sometimes be more difficult for conventionally qualified professionals with their ‘clinical heads on’ (Non-Peer 1, NP1). They believed that being able to relate to what the person at the centre of concern was going through allowed peers to exhibit empathy and form genuine relationships, even with PWLE who were considered hard to engage with:

‘We just find that often clients can relate better to peers. If we are struggling to connect and engage with somebody, often the peer can build some sort of therapeutic relationship. I think it allows them to build trust with the service if they can get on with the peer worker and they may be more open to engaging with the wider team.’ (NP2)

Besides their contribution to network meetings, some peers also offered one-to-one sessions with persons at the centre of concern. The nature of these ranged from a package of appointments to visits on the wards or engagement in practical activities such as shopping, walking or working out. Although peers were aware that such activities are not traditionally associated with Open Dialogue, they were nevertheless more conventionally the sort of domains within which peer workers operated in non-Open Dialogue services. Peers believed they could still maintain a dialogic approach, have meaningful conversations and provide additional or practical support to PWLE who needed it, while engaging with clients in these ways one to one.

### Theme 2: peer practitioners in Open Dialogue opportunities and challenges

#### Valuing the role and its alignment with the ethos of Open Dialogue

Overall, working as a peer practitioner in Open Dialogue was described as a positive experience by participants, who viewed it as a rewarding, enjoyable and meaningful role. While free training was a contributing factor to their involvement in Open Dialogue, peers also viewed the role as an opportunity for growth that would allow them to do meaningful support work:

‘I have loved it, actually. I have loved the closeness of my team, I’m feeling so supported by my colleagues. I just find it, it gives me a great feeling to hear feedback from people that say that it [Open Dialogue] is really helpful.’ (PP5)

Non-peer team members consistently expressed positive perspectives about peer practitioners, describing the experience of working with them as ‘humbling’, ‘connecting’, ‘valuable’ and ‘helpful’. Both peers and others believed that certain attributes of Open Dialogue were aligned with the overall ethos of peer support. In particular, the principle of flattened hierarchy, and Open Dialogue’s emphasis on transparency and empowering individuals, seemed to match the principles and objectives of conventional peer work:

‘I think the modalities complement one another. … There is this sense of the reflective team helping the network and focusing on empowerment which is aligned with peer work. I see from my colleagues how good it feels when they feel that they’ve witnessed somebody achieve something on their own and we’ve just been able to be there to support them as opposed to do for them.’ (PP3)

#### Flattened hierarchy

Having often been through the Open Dialogue training with their colleagues, most peers felt bonded and integrated within their teams. Despite exceptions discussed below, peers reported that a ‘flattened hierarchy’ was achieved in network meetings and most felt respected as equal members of the team when co-facilitating meetings, comfortable with self-disclosure of their lived experience (as formally encouraged by Open Dialogue) and able to challenge established ideas:

‘In my team, there is no doubt that I have a voice to challenge, and I feel safe enough to do so. I am quite vocal with trying to keep the peer values within our team, I think in a way – uniquely for our team – we’ve all become a bit “peer”. I will sit in network meetings and there’s times that I very rarely disclose things and my colleagues are disclosing things that they want to share. That’s felt really good, it’s not felt like the burden to always have to be the one to disclose, we are in equal partnership.’ (PP3)

These positive experiences seemed to be associated with a good understanding of the peer role within their team and management that believed in, and supported, the peer role. Most peers believed their contributions were respected by other members of the team, who largely confirmed this, describing peer practitioners as competent and valuable members of the team and seeing their lived experience as their ‘qualification’ (NP3).

However, some participants shared examples where the principle of flattened hierarchy was not respected. One peer reported exclusion from an initial network meeting on the grounds that only conventionally qualified professionals could conduct clinical assessments. Another peer described another instance of jeopardised flattened hierarchy in which the manager asked the peer not to meet a particular network that was considered potentially triggering and unsafe. Other examples of ‘a compromised flattened hierarchy’ shared by three peers included being spoken for and being ‘outed’ as a peer:

‘… a practitioner had told the person before I’d come in about me [sic] [identified diagnosis] … not taking medication for it and being okay … then this person was rapidly stopping their medication and as I came into the network never having met them, they were relapsing and having a massive episode. And I had real issues around that because it felt like I was responsible for it in some way and I had never met them, never talked to them and I really had a problem with being introduced like that.’ (PP6)

Some peers also expressed their frustration over being paid less than their colleagues who they felt – as Open Dialogue practitioners – were doing the same job, and questioned whether Open Dialogue could fit ‘within a system that puts you on a numbered tier where you’re above or below someone else’ (PP7).

Although these negative experiences led some peers to feel devalued, either by organisational culture or a misinterpretation of the peer role by colleagues, participants generally believed that Open Dialogue offered a favourable context for peer work where their involvement was impactful and respected. Reflecting on experiences of peer work in more conventional mental health services, participants reported feeling unsupported, undervalued and disempowered by diagnosis-led and risk-averse practices that were seen as aligning poorly with the ethos of peer work:

[Describing conventional mental health services] ‘… it felt so shallow and surface level. There is no guidance, there is no support structure, there’s no structure of how you are going to work, it’s challenging, so you kind of do nothing.’ (PP1)

#### Numbers of peer practitioner posts and tokenism

At the time of the interviews with peers, trial sites (with one exception) employed only a single peer practitioner, mostly on a part-time basis. Both peer and qualified practitioner participants reported an overall lack of peer practitioner ‘capacity’ in Open Dialogue teams, resulting in only around a third of clients having a peer involved in their care. While this reflected resource allocation decisions, peer practitioners also reflected upon difficulties in recruiting and retaining peers, which they associated with pay disparities and a lack of career progression for peers:

‘There’s not enough of us I don’t think. But again, who wants to be a peer … who wants to be somewhere where you say, “I’m mad and I get low pay for it and no progression”.’ (PP1)

This raised the question of whether Open Dialogue among these teams could genuinely be considered as POD. Some participants mentioned that it sometimes felt that the peer element was approached as ‘a bit tokenistic’ (PP4), just an ’add-on’ to Open Dialogue that can be ’forgotten’ (PP3) or overlooked:

‘There was this idea that as much as the “P” in the POD is really emphasised in the way POD is spoken about and marketed, in reality in practice peers aren’t really seen as an essential part of each network. Whereas a care coordinator for instance is seen as, “Okay, we need a care coordinator in every network, we don’t need a peer in every network”.’ (PP4),

This participant’s account indicated that, despite the efforts for a flattened hierarchy and the meaningful involvement of peer practitioners in Open Dialogue teams, there was still a sense that peers were not considered essential for the facilitation of network meetings. This was further highlighted by another participant who reported that constant change of management, service restructuring, staff turnover and the intense nature of their team undermined the potential for meaningful involvement in Open Dialogue:

‘You can get a new manager coming in and they’ll have a certain idea about what you should be doing and you just have to like go along with it. Then there’s a restructuring and you get put somewhere else and then and quite often a peer role isn’t really that well defined, and often you are used as a stop gap, you know if they’re short of staff. Peers, you know they are a little bit “other” you know.’ (PP8)

Their account also highlights an opportunistic use of peers who can be othered and ‘used’ as just an additional member of staff, potentially devaluing peers’ lived experience.

### Theme 3: ‘It keeps everyone human’ – peer practitioners’ influence on team and organisational culture

Beyond peer practitioners’ contribution during network meetings, participants also discussed the influence of peers on a wider, team and organisational level. This was discussed in more depth during the interviews with other MDT members who believed that the presence of peers in Open Dialogue teams itself promoted the principle of flattened hierarchy:

‘The first thing that comes to mind is that it makes the conversation a lot more equal, it flattens the hierarchy, that’s a phrase, but there is a truth in that. There’s not a difference, in painting you as the professional – the expert, what have you. It just creates a lot more fluid conversation on an equal footing.’ (NP4)

This was further highlighted by another non-peer practitioner, who believed that the inclusion of peer practitioners in their team ‘keeps everyone human’:

‘I think having a peer in the team makes it a different experience for the clinicians and I think that’s where we are reminded of our humanity in a sense and we’re asked to sort of bring that to the folk that we are working alongside and our way of thinking and being in team meetings.’ (NP5)

Their account supports the idea that, beyond network meetings, peers can impact the way Open Dialogue teams develop working relationships and attend team meetings, perhaps by focusing on the human value beyond professional roles and power structures. A few non-peers specifically mentioned that peers influenced how the remainder of the team used language to refer to PWLE, promoting a more respectful and sensitive approach. By having a colleague with lived experience of mental illness, team members felt they could maintain their focus on supporting people and not ‘reducing them to their diagnosis’ (NP6):

‘You can have discussions in meetings, talking about patients and pejorative things can slip in quite easily … when you have somebody actually who has been through the system themselves in there with you, you know they can help to combat that and they can help keep you grounded in a respectful attitude which values lived experience. I think in terms of the team culture it makes a real difference and even though they are a small part of the team, I think they have an outsized impact on the culture, which I think is really beneficial.’ (NP7)

This was also mirrored in the interviews with peer practitioners. Having often had negative experiences as PWLE, peers valued Open Dialogue as an approach that was more person-centred and empowering. Peers felt that they could support the rest of their teams to retain that focus, avoid labelling and offer prescriptive and manualised care. Participants discussed how they sometimes had to challenge their colleagues when they steered away from the Open Dialogue principles, during either network meetings or team reflective meetings:

‘It helps sort of with “untraining”, I think sometimes, less and less in my own team actually as things progressed, but more towards the beginning I was like “really?” I was surprised that some of my brilliant colleagues just being more mired in thinking about things in terms of risk assessment and safeguarding and systems processes. I think they’ve sometimes maybe not been able to put the person in the centre and I can perhaps bring that in, I step in and remind them of what we should be doing here.’ (PP1)

Non-peer MDT members believed peers were the biggest advocates for Open Dialogue within their teams. Peers’ contribution to team meetings was considered vital in helping teams maintain a dialogic approach and adherence to the principles of the intervention. They described how peers often challenged colleagues when team meetings steered away from the principles of Open Dialogue – for example, when conversations were becoming too focused on clinical aspects of care. This helped teams refocus on reflective practice and emotions that are crucial to dialogic practice:

‘And what’s lovely, when we have supervision, often they [peers] are the ones who bring us back to the feelings, the authenticity of feelings, because they use that so much in their work. So, if we’re going off and being more clinical in our supervision, they will often bring in an authentic conversation about feelings and it can bring us back to what we should be focusing on. So, it’s really, really helpful and if I could, I’d employ more!’ (NP7)

## Discussion

This study examined the experiences of peer practitioners delivering Open Dialogue in the ODDESSI trial, and the perspectives of their colleagues on the work of peers in Open Dialogue teams. Our analysis yielded three themes that explored participants’ experiences and perspectives on the work of peer practitioners in Open Dialogue, and the nature and impact of peer involvement on network meetings and team culture.

Overall, peer practitioners conveyed positive experiences of working in Open Dialogue. They reported enjoying their role and appreciated the ethos of the approach, particularly its focus on empowering individuals and adopting a flattened hierarchy. Indeed, working within a recovery-oriented model such as Open Dialogue, and within an organisational culture aligned with the values of peer work, has been considered essential in the successful implementation of peer work in mental health services^12^ and a predictor of job satisfaction.^26^ In addition, peers reported feeling well integrated within their teams that respected and valued their work, a factor also positively associated with job satisfaction.^11^ Similarly, qualified healthcare professionals reported positive experiences of working with peer practitioners and acknowledged their value within Open Dialogue teams. This can partially be attributed to Open Dialogue training’s intended learning outcomes, including an understanding of the role of peer support and working with peers.^27^

While reflecting on the nature and impact of their work, peer practitioners mostly discussed how sharing their lived experience could support networks. Our findings show that self-disclosure was viewed as a balancing act and seemed to follow an intentional approach to self-disclosure,^28,29^ reflecting on when, why, how and how much to disclose before sharing their lived experiences.^20^ Although self-disclosure has be considered as an emotionally taxing process,^30,31^ participants felt comfortable with it and did not refer to other commonly reported challenges, such as a fear of being stigmatised or appearing less professional to their colleagues.^32^ This can be explained by Open Dialogue being an approach that promotes transparency and self-disclosure by practitioners, regardless of being a peer or not. Consistent with other literature on the topic, both peer and other practitioners believed that self-disclosure helped practitioners develop equal and genuine relationships with networks, instil a sense of hope and open the conversation.^20,28,33,34^

Moreover, our findings indicate that, in addition to self-disclosure, peers’ lived experiences significantly enriched network meetings in various ways. In their conceptual paper, Hendy et al^20^ argue that peer practitioners in Open Dialogue can contribute to dialogic practice through attunement, validation, connection and mutuality. Our findings support this, showcasing how peers’ lived experience allowed them to gain a deeper understanding of PWLE’s emotional states and needs, help them put things into words, adjust their approach to better support them, validate their experiences and offer a sense of normalisation. In a study examining the experiences of PWLE in the Parachute NYC program,^35^ which integrates Open Dialogue and Intentional Peer Support, PWLE reported several benefits of having a peer involved in their care. They reported feeling ’less alienated’ when interacting with peers, appreciated having someone relatable as part of the care team and considered them as a ‘role model’ and a source of hope. Interpreting these findings, we feel that peer practitioners can bring their own ‘expertise’ to network meetings which, like the expertise of conventionally trained Open Dialogue practitioners (e.g. consultant psychiatrists providing information about medication, social workers providing information on housing or employment), can be utilised to complement Open Dialogue.

Beyond their impact on network meetings, our findings suggest that peers also had a broader influence on the overall team culture. Qualified practitioners discussed how peers ‘kept everyone human’, promoting a flattened hierarchy and a more respectful and sensitive use of language. Additionally, both peer and non-peer practitioner participants reported that peers frequently challenged their colleagues when their approach deviated from the principles of Open Dialogue. These findings indicate that, despite the reported limited representation of peers in Open Dialogue teams, they had a disproportionate impact on the overall team culture and adherence to the principles of dialogic practice, which is an important finding in the context of the ODDESSI trial. In a recent qualitative study^36^ about mental health workers’ experiences of working with peers, participants reported similar findings. They believed that peers helped them better understand the needs of PWLE and reflect on the language they used. Most importantly, they believed that peers brought a voice within their teams that was missing and helped them broaden their professional perspectives. Similarly, a report on the impact of peers in mental health services in Ireland showed that service providers believed that peers helped services improve the recovery-orientation of services.^37^ Overall, the impact of peer practitioners on team culture in our study can be conceptualised as a dual process involving both deliberate efforts (e.g. challenging colleagues on their use of language or deviations from Open Dialogue principles) and a symbolic dimension, whereby their very presence fostered a sense of humanity, empathy and inclusion.

However, we must note that it should not be the peer practitioners’ responsibility to foster a recovery-oriented and supportive team culture. Successful integration of peer practitioners in mental health services requires an established supportive organisational culture that values lived experience, or a culture that is sufficiently flexible where leadership can set the direction, implement and manage change.^38,39^ In the context of Open Dialogue specifically, introducing the model to existing mental healthcare settings has been considered challenging, requiring a cultural shift and a transformation at an individual and service level.^22,40,41^ Our findings suggest that the introduction of peers in Open Dialogue teams that have already been educated on the value of peer practitioners and recovery-oriented care could help further facilitate this process of transformation.

### Implications

The integration of peer practitioners into Open Dialogue teams represents a promising adaptation of the approach that can yield benefits for PWLE, peer practitioners themselves and the overall team and service. However, our findings suggest that there is room for improvement when introducing peers in Open Dialogue services, such as ensuring a flattened hierarchy and a respectful approach towards peer practitioners’ lived experiences. Training non-peer staff in understanding the nature and value of peer support, and reflecting on how flattened hierarchy can be ensured, should be a starting point. Moreover, all participants in this study agreed that there was a shortage of peers across all Open Dialogue teams – potentially related to low pay and a lack of structure for progression. Although our findings indicate that even a small representation of peers in Open Dialogue teams can have a substantial impact on the team culture and overall service, the introduction of more peers could allow more networks to have a peer involved in their care. Increased pay for peers who have completed Open Dialogue training, and opportunities for career progression within the peer role, can help overcome recruitment challenges and staff turnover.

### Limitations

One of the main limitations of this study is that data were mainly collected in 2020 and 2021. The experience of both peer and non-peer practitioners could have changed as they became more experienced with Open Dialogue or services further developed. However, our findings still offer a useful description of peer support in ODDESSI POD, and a joint interview with two peers in 2023 did not indicate any significant changes in the way peer support was incorporated in services. Furthermore, although the sample size of peer practitioners in this study could be considered low, it is considerable given the limited number of peers within the ODDESSI trial. Finally, this study used relevant excerpts from interviews with qualified practitioners that had a more general focus on their experience of delivering Open Dialogue. Arguably, interviews that focused solely on their experiences of working with peers could have yielded more nuanced findings.

Our findings indicate that the ethos of Open Dialogue facilitates the introduction of peers in Open Dialogue teams, due to its emphasis on a flattened hierarchy. Open Dialogue represented a platform that allowed meaningful and impactful peer input that could be considered the ‘missing part’ of a recovery-oriented approach. Even in small numbers, peer practitioners in Open Dialogue were found to positively influence network meetings and the overall team culture towards a more empathic and dialogic approach. Despite the overall positive experiences, peers still faced common challenges faced by peers in other services, such as low pay and occasional instances of a compromised flattened hierarchy.

## Supporting information

Anestis et al. supplementary material Anestis et al. supplementary material

## Data Availability

The data that support the findings of this study are available from the corresponding author, T.W., on reasonable request.

## Footnotes

a. We are using the term ‘person/people with lived experience’ (PWLE) throughout the paper in response to recent terminology preferences reports.^8,9^

b. Several terms have been used to refer to people who offer peer support (e.g. peer support workers, peer providers, peer practitioners, peer mentors, peer specialists, experts by experience). For ease of reference, we will use the terms peer and peer worker (or peer practitioner if Open Dialogue trained) interchangeably throughout the paper.

## Supplementary material

The supplementary material is available online at https://doi.org/10.1192/bjo.2025.10833
