# Supplementary material for: Peer-supported Open Dialogue: a qualitative study of peer practitioners’ experiences and non-peer practitioners’ perspectives on peer involvement
Source: BJPsych Open. 2025 Sep 19;11(5):e216. doi: 10.1192/bjo.2025.10833 (PMC12451559; doi:10.1192/bjo.2025.10833)
Supplement: Anestis et al. supplementary material [file S2056472425108338sup001.docx]

**Interview questions for peer practitioners**

- To start with, could you give me a brief idea of your role within the team(s)?
- Can you tell me what your overall experience has been of providing OD care within the ODDESSI trial? (*explore potential positive and negative experiences*)
- Do you think others in your team have a clear sense of your role?
- *Do you believe your team/trust share similar ideas about the purpose of your role? What, if any, differences are there?*
- *Is there anything that could help give team members a clearer understanding of your role? How could this be approached?*
- Are there other peers within your team?
- *How has working with more peers impacted your work?*
- *Are there enough peers within the team or would you benefit from more peers?*
- *What is the benefit of having other peers within your team?*
- Throughout the service user’s care pathway, at what point(s) are you as Peer Practitioners engaged with delivering care?
- *Are Peer Practitioners engaged from the very beginning of a service user’s pathway?*
- *To what extent are you involved in the service user’s care?*
- What would you say is the team’s overall approach to care?
- *How closely do you personally identify with the overall team approach?*
- *How is the team approach reflected in your own practice?*
- *Are there times when the culture of the team/service you are working with means working against your personal principles? If so, could you describe an occasion where this has been the case?*

- If you think of a recent example, can you talk me through what a network meeting might look like?
- *Who is present at these network meetings?*
- *How is the agenda of the meeting organised?*
- *To what extent are the network meetings non-hierarchal?*
- Thinking about core principles of OD, how does the peer role complement these?
  - *Are there any ways in which the peer role supports certain principles of OD, for instance the principal of continuity or the toleration of uncertainty? Or are there any major differences between the peer and the OD model? Could you explain your answer?*
- As you know, to be a peer, one must have lived experience of mental health difficulties. What have been the impacts of disclosing that lived experience?
- *Impact for the service user and the network meeting?*
- *Are there any challenges in self-disclosing with service users?*

**Interview question for non-peer Open Dialogue practitioners**

- *What difference did having a peer practitioner as part of the team make to network meetings and the service users’ care?*
